# Characterization of *POU2F1* Gene and Its Potential Impact on the Expression of Genes Involved in Fur Color Formation in Rex Rabbit

**DOI:** 10.3390/genes11050575

**Published:** 2020-05-20

**Authors:** Naisu Yang, Bohao Zhao, Shuaishuai Hu, Zhiyuan Bao, Ming Liu, Yang Chen, Xinsheng Wu

**Affiliations:** 1 College of Animal Science and Technology, Yangzhou University, Yangzhou 225009, China; dx120180101@yzu.edu.cn (N.Y.); zhao598841633@163.com (B.Z.); 18852726848@163.com (S.H.); 18352764997@163.com (Z.B.); mLiu1994@163.com (M.L.); yangc@yzu.edu.cn (Y.C.); 2 Joint International Research Laboratory of Agriculture & Agri-Product Safety, Yangzhou University, Yangzhou 225009, China

**Keywords:** *POU2F1*, fur color, Rex rabbit, overexpression, RNAi

## Abstract

The naturally colorful fur of the Rex rabbit is becoming increasingly popular in the modern textile market. Our previous study found that POU class 2 homeobox 1 gene (*POU2F1*) potentially affects the expression of genes involved in fur color formation in the Rex rabbit, but the function and regulation of *POU2F1* has not been reported. In this study, the expression patterns of *POU2F1* in Rex rabbits of various colors, as well as in different organs, were analyzed by RT-qPCR. Interference and overexpression of *POU2F1* were used to identify the potential effects of *POU2F1* on other genes related to fur color formation. The results show that the levels of *POU2F1* expression were significantly higher in the dorsal skin of the brown and protein yellow Rex rabbits, compared with that of the black one. *POU2F1* mRNAs were widespread in the tissues examined in this study and showed the highest level in the lungs. By transfecting rabbit melanocytes with an *POU2F1*-overexpression plasmid, we found that the POU2F1 protein was located at the nucleus, and the protein showed the classic characteristics of a transcription factor. In addition, abnormal expression of *POU2F1* significantly affected the expression of pigmentation-related genes, including *SLC7A11*, *MITF*, *SLC24A5*, *MC1R*, and *ASIP*, revealing the regulatory roles of *POU2F1* on pigmentation. The results provide the basis for further exploration of the role of *POU2F1* in fur color formation of the Rex rabbit.

## 1. Introduction

Rex rabbit fur is becoming a fashionable raw material for modern textiles and has outstanding features, including aesthetic appeal, smoothness, softness, lightness, flexibility, and heat-retaining properties. The multiple natural colors of the Rex rabbit have made it increasingly popular. In particular, some special colors, such as chinchilla, brown, protein yellow, and protein chinchilla, have the advantages of being not only attractive but also nontoxic and ecological, as no artificial dyes are required [1]. The huge demand in the market for fashionable Rex rabbit fur has promoted research into the characteristics of its fur color.

The formation of fur color in mammals is a complex process influenced by diverse factors, including the environment, management, and the genetic background [2,3]. Briefly, the relative quantity and distribution of melanocyte products, such as eumelanin and pheomelanin, determine the fur color [4,5]. It has been reported that gene expression affects the pigmentation of rabbit fur through various mechanisms; for example, some genes, such as *SLC7A11* [6,7,8], melanocortin receptor 1 (*MC1R*) [9,10,11], microphthalmia-associated transcription factor (*MITF*) [12], and *ASIP* [13], are likely to influence the production and deposition of melanin.

Recently, *SLC7A11* was found to be modulated by POU class 2 homeobox 1 gene (*POU2F1*) in rabbit pigmentation [14]. *POU2F1*, also known as *OCT1*, *OTF1*, or *oct-1B* [15], is a potent regulator of stress responses, metabolism, and tumorigenicity and is itself regulated by phosphorylation, ubiquitination, O-GlcNAcylation, and other mechanisms [16]. Recent studies into *POU2F1* have focused on its impacts on cancers and tumors [17,18,19,20], especially hepatocellular carcinoma [21,22]. Furthermore, *POU2F1* was identified as a transcription factor that binds to the promoter region of *SLC7A11* via two binding sites in the Rex rabbit [14]. However, little is known about the role of *POU2F1* in fur color formation in mammals, and the effects of the gene on the fur color of the Rex rabbit remain unclear. 

Therefore, this study is the next stage of our research to analyze the potential impacts of *POU2F1* on the significant genes involved in the formation of Rex rabbit fur color. The expression pattern of *POU2F1* in the dorsal skin of the Rex rabbit with different fur colors, and in different organs, was analyzed by RT-qPCR. Additionally, a pcDNA3.1(+)-Myc-*POU2F1* vector and siRNAs were constructed to analyze the potential regulatory roles of *POU2F1* on *SLC7A11*, as well as some other pigmentation-related genes, such as *SLC24A5*, *MITF*, *MC1R*, *CREB1*, and *ASIP*, to reveal the regulatory role of *POU2F1* in fur color formation of the Rex rabbit.

## 2. Materials and Methods 

### 2.1. Animals and Samples

Eighteen 6-month-old Rex rabbits with 6 different fur colors (*n* = 3 for each color), including black (BL), chinchilla (CH), white (WH), brown (BR), protein yellow (PY), and protein chinchilla (PC), were provided by a rabbit breeding farm in Yuyao, Zhejiang, China. A 1 cm^2^ sample of dorsal skin tissue was collected from Rex rabbits of each color (*n* = 3) after anesthesia by injection of sodium pentobarbital solution (0.7%) into the ear vein. Tissue samples of different organs (heart, liver, spleen, lung, kidney, jejunum, colon, ileum, cecum, rectum, dorsal skin, sacculus rotundus, and gizzard) were collected from other white and black Rex rabbits (*n* = 3, respectively). White is the most common color of Rex rabbit and is widely used for fur production around the world because it is easily dyed and has good plasticity, while black rabbits were chosen for their striking contrast. These rabbits were euthanized by ear vein injection of 25 mL air after deep anesthesia, and organ samples were collected about 5 min after confirmation of the absence of heartbeat and death. All tissue samples were placed in liquid nitrogen immediately after being cut into small pieces and stored at −80 °C until use. The experimental procedures were approved by the Animal Care and Use Committee of Yangzhou University (Yangzhou, China, 24 October 2017, No. 201710001).

### 2.2. Melanocyte Culture and RNA Extraction

Melanocytes were separated by two-step enzymatic digestion from a 1.5 × 1.5 cm^2^ section of dorsal skin from white Rex rabbits according to our previous report [23]. Total RNA of the dorsal skin and organs was extracted using RNAsimple Total RNA kit (Tiangen, Beijing, China), and total RNA from melanocytes was extracted using Trizol reagent (Invitrogen, Carlsbad, CA, USA), according to the manufacturer’s instructions. Electrophoresis with 1% agarose gel was used to monitor RNA degradation and contamination. RNA purity and concentration were measured using a NanoPhotometer spectrophotometer (Thermo Fisher Scientific, Wilmington, NC, USA).

### 2.3. Real-Time qPCR

The dorsal skin and organs were submitted for quantitative real-time PCR to detect the expression levels of *POU2F1*. Briefly, cDNA was synthesized from approximately 1 μg RNA using HiScript II Q Select RT SuperMix for qPCR (Vazyme, Nanjing, China). Then, the cDNA was used for RT-qPCR with the AceQ qPCR SYBRR Green Master Mix (Vazyme, Nanjing, China), based on the manufacturer’s instructions. To improve the data reliability, cDNA of each sample was tested three times for technical replication, and three biological replicates were tested for each group. In order to prevent deviation due to possible unstable expression of reference genes, glyceraldehyde 3-phosphate dehydrogenase (*GAPDH*), β-*actin*, and hypoxanthine phosphoribosyltransferase 1 (*HPRT1*) were used as reference genes [24]. The primer sequences for RT-qPCR are listed in Table 1. The output data were analyzed using QuantStudioR 5 (Applied Biosystems), and the expression levels were analyzed according to the 2^−∆∆Ct^ method [25].

### 2.4. Construction of pcDNA3.1(+)-Myc-POU2F1 Vector

**Figure 1:**
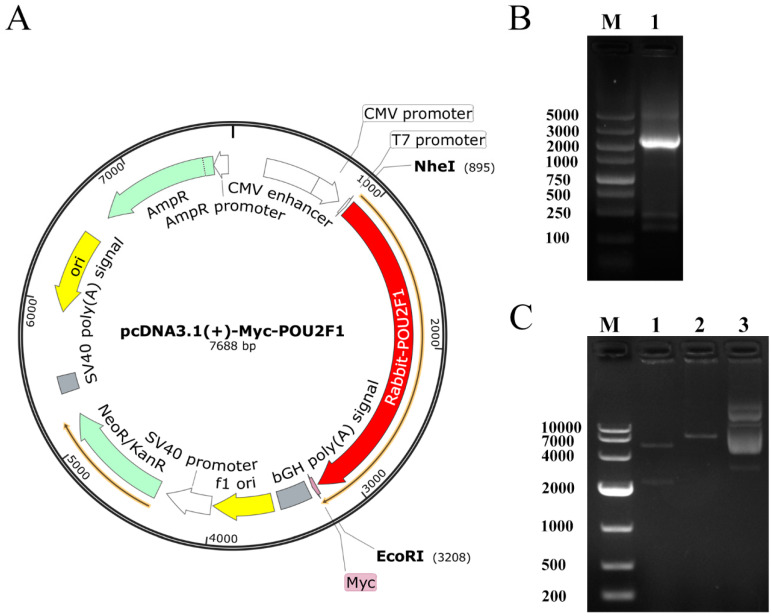
Identification and construction of pcDNA3.1(+)-Myc-*POU2F1*. (**A**) Plasmid map of pcDNA3.1(+)-Myc-*POU2F1*. The gene sequence encoding POU2F1 was inserted into the pcDNA3.1(+)-Myc vector between the corresponding restriction sites NheI and EcoRI. (**B**) PCR products of *POU2F1* gene. M, DL5000 DNA Marker, Lane 1, *POU2F1* mRNA sequence. (**C**) Identification of pcDNA3.1(+)-Myc-*POU2F1* digested by NheI and EcoRI. M, DL10000 DNA Marker. Lane 1, production of pcDNA3.1(+)-Myc-*POU2F1* after NheI and EcoRI digestion. Lane 2, production of pcDNA3.1(+)-Myc-*POU2F1* after EcoRI digestion. Lane 3, pcDNA3.1(+)-Myc-*POU2F1* before digestion.

The PrimeScript™ 1st Strand cDNA Synthesis Kit (Takara, Beijing, China) was used to synthesize Rex rabbit skin cDNA of high quality for the construction of the *POU2F1* overexpression vector. The CDS sequence of the *POU2F1* gene was amplified by PCR using Phanta Max Super-Fidelity DNA Polymerase (Vazyme, Nanjing, China), according to the mRNA sequence of rabbit *POU2F1* (NC_013681.1). The amplified CDS sequence of *POU2F1* was subcloned into an NheI- and EcoRI-digested pcDNA3.1(+)-Myc vector (Invitrogen) (Forward primer: gggagacccaagctggctagcATGGCGGACGGAGGAGCA; Reverse primer: gtagtcggatcctttgaattcCTGTGCCTTGGAGGCGGT), and the recombinant plasmid was named pcDNA3.1(+)-Myc-*POU2F1* (Figure 1) for the subsequent steps. The pcDNA3.1(+)-Myc-*POU2F1* was transferred into melanocytes to overexpress the *POU2F1* gene, and the expression levels of the fur-color-related genes (*SLC24A5*, *SLC7A11*, *MITF*, *MC1R*, *CREB1*, and *ASIP*) were detected by RT-qPCR, according to the above method.

### 2.5. Subcellular Localization of POU2F1 Protein

PSORT (www.psort.org) was used to predict the localization of the POU2F1 protein. The pcDNA3.1(+)-Myc-*POU2F1* was transferred into melanocytes using ExFect 2000 (Vazyme, Nanjing, China), according to the manufacturer’s instructions with 1 μg pcDNA3.1(+)-Myc-*POU2F1* in 1 μL ExFect 2000 for each well, and cultured in a 24-well plate with CO_2_ at 37 °C for 24 h. Then, 4% paraformaldehyde was used to fix the cells at room temperature (RT) for 20 min, followed by penetration in 0.3% Triton X-100 (Solarbio, Beijing, China) at RT for 10 min and blocking in goat serum at 37 °C for 30 min. The cells were incubated with the primary antibody (Myc) at 4 °C overnight and with the second antibody (Cy3-conjugate) at RT for 2 h. After dying in DAPI for 10 min, the cells were observed and photographed under a fluorescent inverted microscope. The cells were washed with PBS (three times for 5 min/time) between each of the above steps.

### 2.6. Inhibition of POU2F1 by siRNA

Three siRNAs (with 5′ FAM modification) and negative control siRNAs were ordered from Suzhou GenePharma Co., Ltd. (Suzhou, China) (Table 2). The siRNAs were diluted into 20 μM with DEPC water, and then the complexed 2 μL diluted siRNAs and 1 μL Lipofectamine 2000 (InvitrogenTM) were transferred to the melanocytes when the cell confluence reached approximately 70%. After 24 h, fluorescence microscopy was used to detect the transfection efficiency. The total RNA extracted from the cells after transfection was used for RT-qPCR to examine the expression levels of *POU2F1* and other genes, according to the above methods.

### 2.7. Statistical Analysis

All values in the text and figures are presented as the means ± standard deviation (SD) calculated by GraphPad Prism software (GraphPad Software, La Jolla, CA, USA). A paired T-test from SPSS 25 was performed between 2 groups (control and each test group). Statistical differences are presented at probability levels of *p* < 0.05 (*), *p* < 0.01 (**), and *p* < 0.001 (***).

## 3. Results

### 3.1. POU2F1 Gene Expression in Dorsal Skin of Rex Rabbits with Different Fur Colors

**Figure 2:**
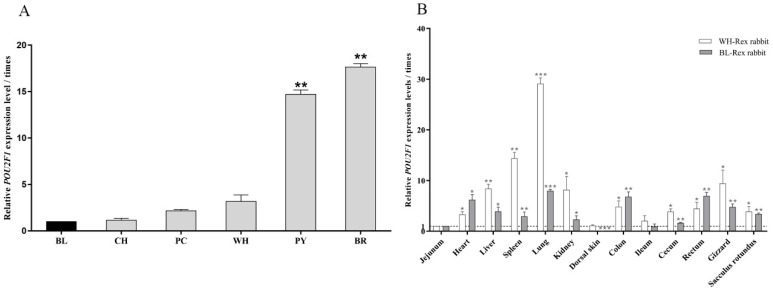
Expression patterns of *POU2F1* gene. (**A**) The expression level of *POU2F1* in the dorsal skin of Rex rabbits with different fur colors. BL: black, CH: chinchilla, WH: white, BR: brown, PY: protein yellow, and PC: protein chinchilla. *POU2F1* expression levels in the dorsal skin of the black Rex rabbit were used as the control to calculate the relative expression levels in Rex rabbits of other colors in figure A. (**B**) The expression level of *POU2F1* in different organ tissues of the white and black Rex rabbit. *POU2F1* expression levels in jejunum of the white and black Rex rabbit were used as controls for calculating the relative expression level in corresponding Rex rabbits in figure B. * *p* < 0.05, ** *p* < 0.01, and *** *p* < 0.001.

The expression levels of *POU2F1* in the dorsal skin of Rex rabbits with different fur colors were detected by RT-qPCR. As shown in Figure 2A, *POU2F1* was generally expressed in the dorsal skin of Rex rabbits of each color but showed significantly different expression levels (*p* < 0.05). *POU2F1* had the lowest expression level in the BL-Rex rabbit, which was used as a control to calculate the fold change of *POU2F1* gene expression in Rex rabbits with other fur colors. The highest expression levels of *POU2F1* were seen in the BR-Rex rabbit (15.63 times), followed by the PY-Rex rabbit (12.37 times), which were significantly higher than that in Rex rabbits with the other four fur colors (*p* < 0.05). There was no significant difference in *POU2F1* expression between CH, PC, and WH-Rex rabbits, in which *POU2F1* expression was generally low at around 1–3 times the levels in the BL-Rex rabbit.

### 3.2. The Tissue Distribution of POU2F1 Gene Expression in WH- and BL-Rex Rabbits

*POU2F1* expression in different organs of the WH- and BL-Rex rabbits is shown in Figure 2B. The relative expression levels of *POU2F1* in different organs of BL-Rex rabbits normalized by three reference genes (*GAPDH*, β-*actin* and *HPRT1*) were generally consistent (Supplementary Figure S1). *POU2F1* expression in the jejunum, which had a relatively low and stable expression level in BL- and WH-Rex rabbits, was used as a control (defined as 1) to calculate the fold change in *POU2F1* expression in other organs. *POU2F1* was widely expressed in different organs of the WH- and BL-Rex rabbits, and almost all other organs (except dorsal skin and ileum) had significantly higher expression levels than jejunum (*p* < 0.05). In addition, *POU2F1* showed the highest expression levels in the lung in both WH- and BL-Rex rabbits (29.15 and 7.99 times higher, respectively), while the dorsal skin of BL-Rex rabbit had significantly lower *POU2F1* expression (0.06 times).

### 3.3. Subcellular Localization of POU2F1 Protein

**Figure 3:**
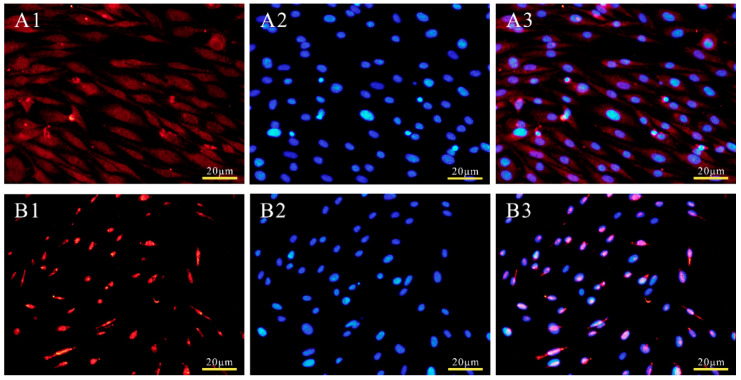
Subcellular localization of POU2F1 protein in rabbit melanocytes. The pcDNA3.1(+)-Myc (**A1**–**A3**) and recombinant plasmids pcDNA3.1(+)-Myc-*POU2F1* (**B1**–**B3**) were transiently transfected into rabbit melanocytes. Cells in groups A and B are melanocytes magnified 40 times by fluorescence inverted microscope. **A1** (**B1**) is the Myc-tag dyed by Cy3, and **A2** (**B2**) is the DAPI-dyed nucleus, which are merged in **A3** (**B3**).

PSORT predicted that 95.7% of the POU2F1 protein would be located at the nucleus. Subcellular localization analysis was used to determine the localization of the POU2F1 protein. As shown in Figure 3A, the empty pcDNA3.1(+)-Myc vector without the *POU2F1* gene was randomly distributed throughout the whole cell, including the nucleus and cytoplasm. The pcDNA3.1(+)-Myc-*POU2F1* was only found in the nucleus (Figure 3B), indicating that the localization of the POU2F1 protein in the dorsal skin cells of Rex rabbits was nuclear, which is consistent with the prediction.

### 3.4. The Effects of POU2F1 Gene on Other Pigmentation-Related Genes

**Figure 4:**
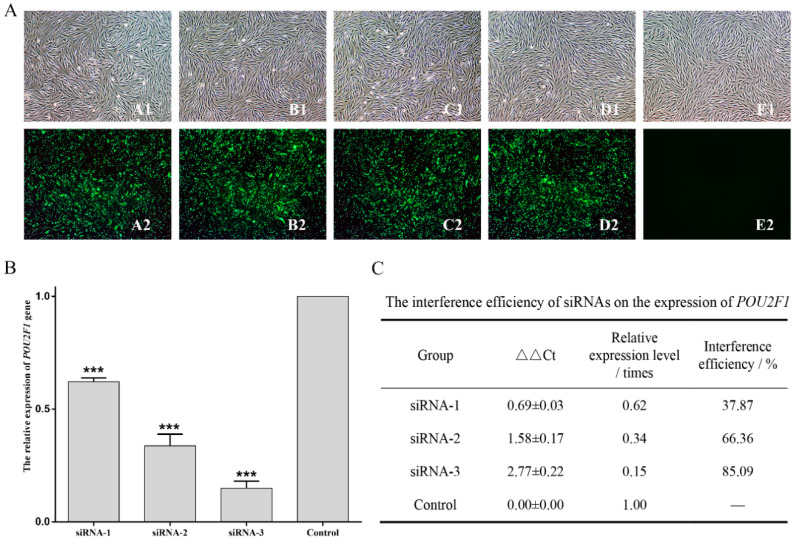
Interference efficiency of siRNAs on *POU2F1* expression. (**A**) Melanocyte morphology 24 h after FAM-siRNAs transfection. (**A**–**E**) represent siRNA-1, siRNA-2, siRNA-3, NC, and blank, respectively. (**B**,**C**) show interference efficiency of siRNAs on *POU2F1* expression. *** *p* < 0.001.

*POU2F1* siRNA interference and overexpression were performed in melanocytes to analyze the potential functional mechanisms of *POU2F1* during melanin deposition in the dorsal skin of Rex rabbits, followed by detection of the expression levels of pigment-related genes (*SLC7A11*, *SLC24A5*, *MITF*, *MC1R*, *CREB1* and *ASIP*) by RT-qPCR. siRNA1, siRNA2, and siRNA3 were successfully transferred into melanocytes (Figure 4A); siRNA3 had the best interference efficiency (85.09%) (Figure 4B,C) and was used for *POU2F1* interference in the next stage of the experiment. 

**Figure 5:**
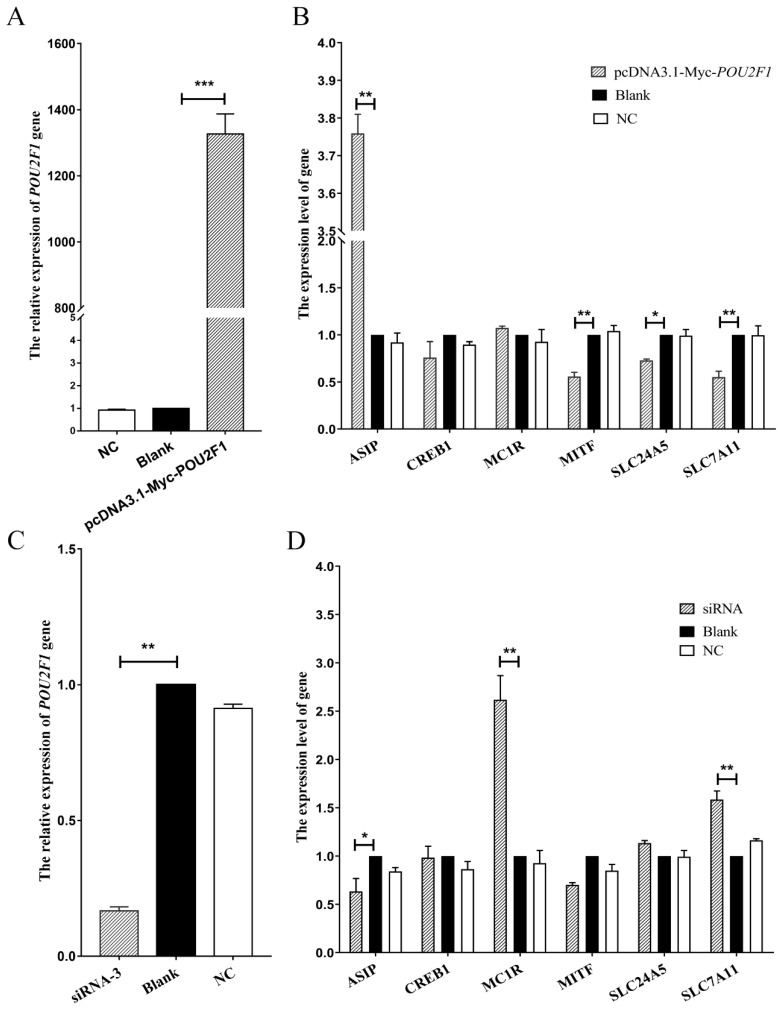
Expression levels of *SLC7A11* and other pigmentation-related genes after the overexpression and interference of *POU2F1*. (**A**,**C**) are the relative gene expression levels of *POU2F1* in rabbit melanocytes transfected with pcDNA3.1(+)-Myc-*POU2F1* vector and siRNA-3, respectively; (**B**,**D**) are the relative expression levels of other pigmentation-related genes in rabbit melanocytes transfected with pcDNA3.1(+)-Myc-*POU2F1* vector and siRNA-3, respectively. The relative expression levels of the above genes were measured by RT-qPCR and calculated by the 2^−∆∆Ct^ method. * *p* < 0.05, ** *p* < 0.01, and *** *p* < 0.001.

The *POU2F1* gene was overexpressed by 1326.3 times the baseline by pcDNA3.1(+)-Myc-*POU2F1* (Figure 5A). Simultaneously, the *SLC7A11* gene was significantly downregulated 0.55 times (Figure 5B), indicating the negative regulation of the *SLC7A11* gene by *POU2F1*. In addition, *MITF* and *SLC24A5* were downregulated (0.56 and 0.73 times, respectively), but *ASIP* was significantly upregulated 3.76 times. However, when *POU2F1* expression was inhibited by siRNA3 (Figure 5C), the *SLC7A11* gene showed significant overexpression at 1.58 times the baseline (Figure 5D), emphasizing the inhibitory role of *POU2F1* on *SLC7A11*. In addition, *ASIP* was significantly downregulated 0.63 times, and *MC1R* was significantly upregulated 2.62 times.

## 4. Discussion

In this study, we compared the expression levels of *POU2F1* in Rex rabbits of various colors to investigate the possible impacts of the gene on fur color. *POU2F1* was generally but differently expressed in the dorsal skin of Rex rabbits, revealing its potential roles in fur color formation. *POU2F1* showed the lowest expression levels in BL-Rex rabbits and the highest expression levels in the BR-Rex rabbit. In addition, the opposite pattern of expression of the *SLC7A11* gene was identified in Rex rabbits with the same color [14]. This is consistent with the negative regulation of the *SLC7A11* gene by *POU2F1*, which was reported previously. Additionally, *SLC7A11* was reported to positively regulate melanin levels, and higher *SLC7A11* gene expression levels led to increased melanin deposition [14]. This may explain why *POU2F1* and *SLC7A11* showed the highest and lowest expression levels, respectively, in BL-Rex rabbits.

Additionally, the tissue distribution of *POU2F1* expression was analyzed to explore potential differential expression. Because the use of *GAPDH* as a reference gene to correct gene expression levels in the Rex rabbit was relatively novel, another two reference genes (β-*actin* and *HPRT1*) were employed for the detection of *POU2F1* expression levels. The expression levels of *POU2F1* normalized by *GAPDH*, *HPRT1* and β-*actin* were roughly consistent, which revealed the usability of *GAPDH* as a reference gene in the Rex rabbit and improved the reliability of the data in this study. *POU2F1* was widely expressed in different organs of white and black Rex rabbits, with the highest expression levels in the lungs. *POU2F1* has been reported to be involved in carcinogenesis and other disease processes by promoting cell proliferation and metastasis [26], e.g., in type 2 diabetes [15,27], head and neck cancer [28], cervical cancer [19], and liver cancer [29]. The wide involvement of *POU2F1* in various kinds of cancer and in the immune response to cancers suggests its general expression in different organs, which was also found in this study. Interestingly, most recent studies on the *POU2F1* gene have focused on its regulatory impacts on liver cancer [21,22,29]. *POU2F1* was found to be highly expressed in the liver of Rex rabbits, suggesting a potential immune-related role for the *POU2F1* gene, a phenomenon which deserves further research. In addition, *POU2F1* showed the highest expression levels in the lungs, which may be related to the expression and important roles of *SLC7A11* in response to lung cancer [30,31].

As a transcription factor, *POU2F1* was predicted to be expressed and to have some biological functions in the nucleus of melanocytes of the Rex rabbit, as was reported in human HEK-293T cells [32]. A subcellular localization study confirmed the prediction that the POU2F1 protein is located at the nucleus and does not have a transmembrane structure, which are classical characteristics of transcription factors. *POU2F1* played biological roles in the nucleus of melanocytes, suggesting it has a potential role as a transcription factor regulating the expression of some other genes. Overexpression and interference of *POU2F1* revealed its negative regulatory effect on the *SLC7A11* gene, which was consistent with the findings of our previous study. Abnormal expression of *POU2F1* not only regulated the expression of the *SLC7A11* gene but also some other fur-color-related genes. The positive influence of *POU2F1* on the expression of *ASIP* was also identified in this study, which fits with the negative relationship between *ASIP* and *SLC7A11* [14]. In addition, expression of the *MC1R* gene was upregulated when *POU2F1* expression was affected. Both *ASIP* and *MC1R* have been reported to be involved in the melanogenesis pathway and the regulation of fur color formation [9,10,13]. Furthermore, the overexpression of *POU2F1* negatively regulated the expression of *SLC24A5* and *MITF*, which are related to pigmentation. The findings of various studies indicate the important role *SLC24A5* has on human skin pigmentation: it regulates human epidermal melanogenesis and related diseases, such as nonsyndromic oculocutaneous albinism [33,34,35]. *MITF* plays significant roles in signal transduction and transcription in melanocytes during the color formation of skin and fur [36,37]. In addition, *ASIP*, *MC1R*, *SLC24A5,* and *MITF* were found to not only affect the formation of human skin color [38,39] but also affect fur color in different animals, including mice [37], sheep [40,41], horses [42,43,44], pigs [45], and rhesus macaques [46]. The significant associations between *POU2F1* and other fur-color-related genes further revealed the potential impacts of *POU2F1* on Rex rabbit fur color.

## 5. Conclusions

The *POU2F1* gene was significantly more highly expressed in the dorsal skin of brown and protein yellow Rex rabbits compared with that of Rex rabbits with black, chinchilla, white, and protein chinchilla fur. *POU2F1* affects the expression of genes, including *SLC24A5*, *SLC7A11*, *MITF*, *MC1R*, and *ASIP*, that are related to the formation of fur color in Rex rabbits. This study highlighted the potential regulatory role of *POU2F1* in the fur color of Rex rabbits.

## Figures and Tables

**Table 1:** Primer sequences for RT-qPCR.

Genes	F/R	Sequences of Primers (5′-3′)	Accession No.
*MITF*	F	AGCTTGCCATGTCCAAACCAG	XM_008260927.1
	R	TTCATACTTGGGCACTCGCTCT	
*MC1R*	F	CCTCGGCACCCCACTTGCAG	NW_003159591.1
	R	CAGCACCTCCTTGAGCGTCCTG	
*SLC24A5*	F	CTGTCCCTCATGAAGACTACCGTT	XM_002717863.2
	R	ACACCACCTATGTTCAACTGGC	
*ASIP*	F	CTGTGCTTCCTCACTGCCTATAGCC	NM_001122939.1
	R	TTCAGCGCCACAATGGAGACCGAA	
*CREB1*	F	CCTCCCCAGCACTTCCTACACA	XM_008259050.1
	R	TTCAGCTCCTCAATCAGCGTCT	
*SLC7A11*	F	TCACCATTGGCTACGTGCT	NC_013683.1
	R	GCCACAAAGATCGGAACTGCT	
*POU2F1*	F	GCAGCAGCAGCAGATTCAAGAATG	XM_017345713.1
	R	GTGTGCCTGTGTTGCCATCTCC	
*GAPDH*	F	CACCAGGGCTGCTTTTAACTCT	NM_001082253.1
	R	CTTCCCGTTCTCAGCCTTGACC	
β-*actin*	F	CGGCACCAGGGCGTGAT	NM_001101683
	R	CGCTTGCTCTGGGCCTCGT	
*HPRT1*	F	CGTCGAGGACTTGGAAAGGG	NM_001105671.1
	R	TTGAGCACACAGAGGGCTAC	

F, forward primers; R, reverse primers.

**Table 2:** Primer sequences of siRNAs.

siRNAs	S/A	Sequences of Primers (5′-3′)
siRNA1	S	GGAAGAGCCCAGUGACCUUTT
	A	AAGGUCACUGGGCUCUUCCTT
siRNA2	S	CCUUGAACCUCAGCUUUAATT
	A	UUAAAGCUGAGGUUCAAGGTT
siRNA3	S	GCCUCCACCUCCGAGACAUTT
	A	AUGUCUCGGAGGUGGAGGCTT

S, sense strands; A, anti-sense strands.

## Supplementary Materials

The following are available online at https://www.mdpi.com/2073-4425/11/5/575/s1, Figure S1: Expression level of POU2F1 in different organ tissues of Black Rex rabbit.
