# Supplementary material for: Characterization of POU2F1 Gene and Its Potential Impact on the Expression of Genes Involved in Fur Color Formation in Rex Rabbit
Source: Genes (Basel). 2020 May 20;11(5):575. doi: 10.3390/genes11050575 (PMC7288328; doi:10.3390/genes11050575)
Supplement: Supplementary file 1 [file genes-11-00575-s001.pdf]

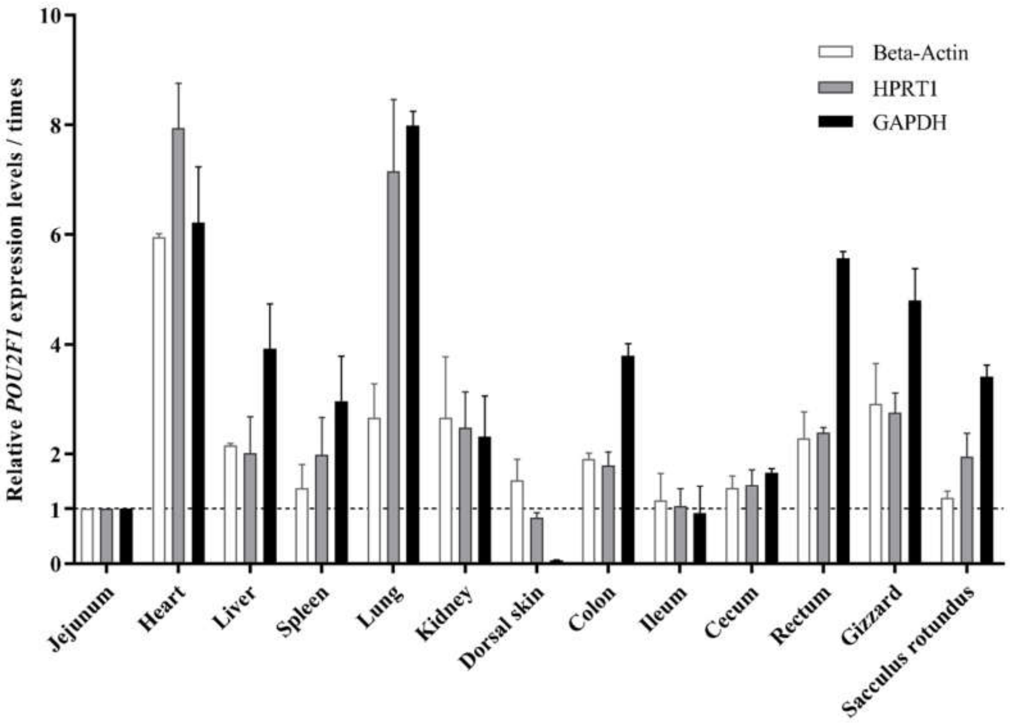

Supply Figure 1. Expression level of *POU2F1* in different organ tissues of Black Rex rabbit. *POU2F1* expression in the jejunum was used as the control to calculate the relative expression fold changes of *POU2F1* in other organ tissues. White, gray, and black bar were the *POU2F1* expression level normalized by *Beta-actin*, *HPRT1*, and *GAPDH* respectively.
